# Rapid entomological assessment in eight high malaria endemic regencies in Papua Province revealed the presence of indoor and outdoor malaria transmissions

**DOI:** 10.1038/s41598-024-64958-w

**Published:** 2024-06-25

**Authors:** Ismail E. Rozi, Dendi H. Permana, Lepa Syahrani, Puji B. S. Asih, Siti Zubaidah, Rifqi Risandi, Suradi Wangsamuda, Farahana K. Dewayanti, Michael R. Demetouw, Silas Mabui, Marthen M. F. Robaha, Maria E. Sumiwi, Michael J. Bangs, Neil F. Lobo, William A. Hawley, Din Syafruddin

**Affiliations:** 1 Eijkman Research Center for Molecular Biology, National Research and Innovation Agency (BRIN), Cibinong, Indonesia; 2 Doctoral Program in Faculty of Medicine, Hasanuddin University, Makassar, Indonesia; 3 Doctoral Program in Biomedical Sciences Faculty of Medicine, University of Indonesia, Jakarta, Indonesia; 4 Doctoral Program in Faculty of Mathematics and Natural Sciences, University of Indonesia, Depok, Indonesia; 5 Papua Province Health Office, Jayapura, Papua Indonesia; 6 United Nations International Children’s Emergency Fund (UNICEF), Jakarta, Indonesia; 7 PT Freeport Indonesia, International SOS, Freeport Medical Services, Kuala Kencana, Papua Indonesia; 8 Department of Entomology, Faculty of Agriculture, Kasetsart University, Bangkok, Thailand; 9 Eck Institute for Global Health, University of Notre Dame, Indiana, USA; 10 Department of Parasitology, Faculty of Medicine, Hasanuddin University, Makassar, Indonesia; 11 Hasanuddin University Medical Research Center (HUMRC), Makassar, Indonesia

**Keywords:** Entomological assessment, Human landing catch, Larval site surveillance, Papua Province, Indonesia, Diseases, Health care

## Abstract

Malaria in eastern Indonesia remains high despite significant reduction and elimination in other parts of the country. A rapid entomological assessment was conducted in eight high malaria endemic regencies of Papua Province, Indonesia, to expedite malaria elimination efforts in this region. This study aims to characterize specific, actionable endpoints toward understanding where and when malaria transmission is happening, where interventions may function best, and identify gaps in protection that result in continued transmission. The entomological assessment included identifying potential vectors through human landing catch (HLC), indoor morning and night resting collections, identification of larval sites through surveillance of water bodies, and vector incrimination toward understanding exposure to malaria transmission. Human landing catches (HLCs) and larval collections identified 10 *Anopheles* species, namely *Anopheles koliensis*, *Anopheles punctulatus*, *Anopheles farauti*, *Anopheles hinesorum*, *Anopheles longirostris*, *Anopheles peditaeniatus*, *Anopheles tesselatus, Anopheles vagus, Anopheles subpictus* and *Anopheles kochi*. The most common and abundant species found overall were *An. koliensis* and *An. punctulatus*, while *An. farauti* was found in large numbers in the coastal areas of Mimika and Sarmi Regencies. Vector incrimination on *Anopheles* collected from HLCs and night indoor resting demonstrated that *An. koliensis* and *An. punctulatus* carried *Plasmodium* in Keerom, Jayapura, and Sarmi Regencies. Analysis of HLCs for the most common species revealed that the *An. koliensis* and *An. punctulatus,* bite indoors and outdoors at equal rates, while *An. farauti* predominantly bite outdoors. Larval surveillance demonstrated that most water bodies in and surrounding residential areas contained *Anopheles* larvae. This study demonstrated indoor and outdoor exposure to mosquito bites and gaps in protection, enabling exposure to infectious bites in all regencies. This explains why current malaria control efforts focusing on indoor protection have failed to substantially reduce malaria incidence in the region. Optimization of insecticide-treated bed nets (ITNs), as well as installment of mosquito screens in houses, may further reduce indoor transmission. For outdoor transmission, the use of community-centric approaches to reduce or eliminate larval sources within and surrounding the village through the guidance of locally stationed entomologists, along with Social and Behavior Change mediated health education towards the local adoption of mosquito protection tools during outdoor activities, may reduce malaria transmission.

## Introduction

Indonesia is a tropical archipelago with 16,772 islands covering a land area of 1.89 million km^2^, a water area of 3.26 million km^2^, and a population of over 273 million people. Malaria has consistently remained a public health problem in Indonesian Papua. Although concerted efforts by the Ministry of Health have successfully reduced malaria incidence to less than half—from 2010 through 2015 (the malaria elimination program was launched in 2009), efforts to reduce malaria cases have remained stagnant since^1–3^, particularly in Papua. Indonesia reported a malaria Annual Parasite Incidence (API) of 0.93 at the time of this study (2019), where the three highest provinces’ APIs, 64.05, 7.38, and 2.37, came from Papua, Papua Barat, and Nusa Tenggara Timur, respectively. Despite only 1.5% of the country’s population, Papua Province has 74% of malaria cases. While 300 regencies (58.4%) in the country have been certified as free from malaria transmission, regencies in Papua face the most complex challenges in achieving the goal of malaria elimination by 2030, considering their current very high endemicity status^4^. Therefore, extraordinary efforts and the well-managed implementation of malaria control interventions are required to achieve this goal.

**Figure 1:**
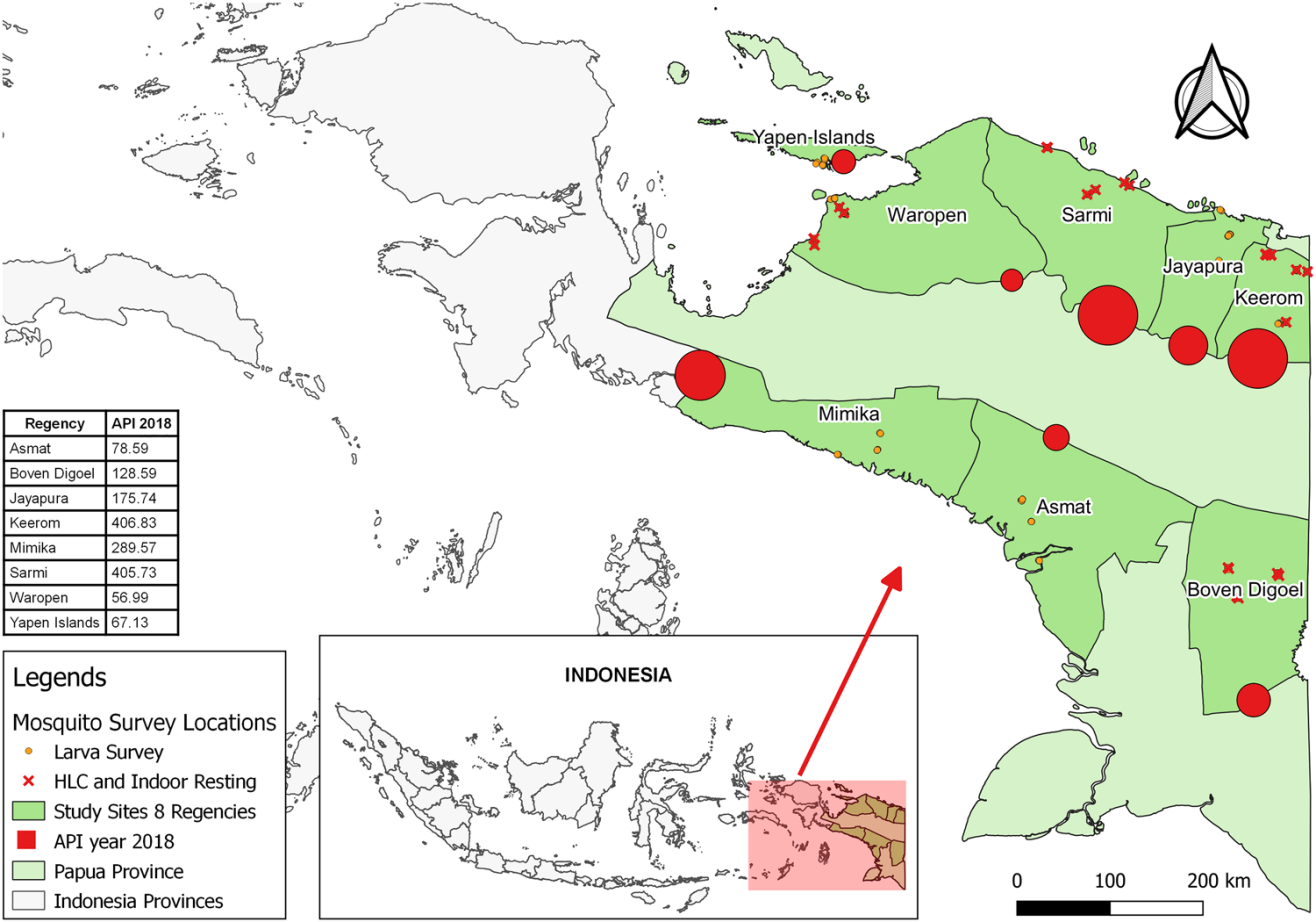
Location of eight regencies in Papua Province: Keerom, Mimika, Jayapura, Sarmi, Boven Digul, Yapen Islands, Waropen, and Asmat, with the highest Annual Parasite Incidence (API) of malaria in 2018 (red dot areas scaled eight districts’ API values).

**Figure 2:**
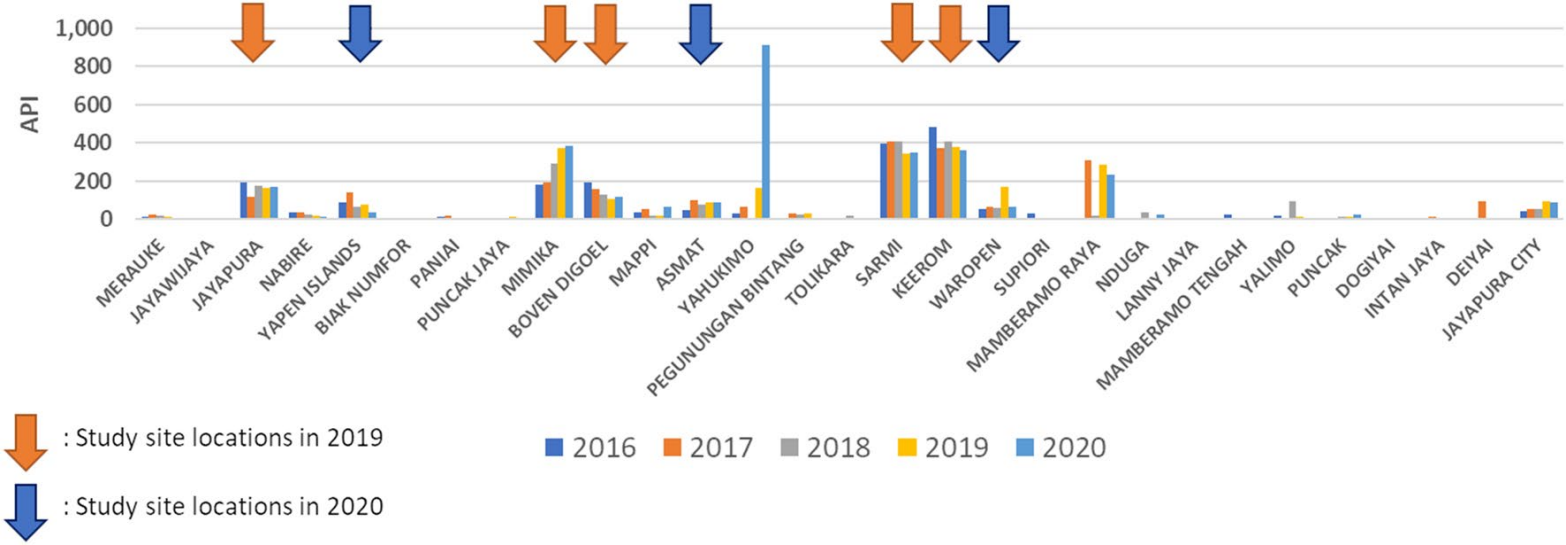
Annual parasite incidence (API) per 1000 population in districts of Papua Province between 2016 and 2020 (Data source: Papua Province Health Office).

Papua, located east of Wallace’s Line, has endemic vectors not found in western Indonesia. Some of these species may be more anthropophilic than those found in the west; further, Papua has no pronounced dry season, leading to a persistent perennial transmission^5^. Five regencies in Papua—Keerom, Mimika, Jayapura, Sarmi, and Boven Digul, reported the highest APIs—greater than 100 per thousand population in 2018. Meanwhile, three districts, Yapen Islands, Waropen, and Asmat, showed APIs of 50–100 per thousand population (Fig. 1). By the end of 2020, the total malaria cases in these eight regencies were still high (Fig. 2)^6,7^.

Although the Ministry of Health has advocated the strengthening of malaria control efforts in Papua by providing effective antimalarial drugs, together with training in malaria diagnosis, provision of long-lasting insecticide-treated mosquito nets (LLINs) to pregnant women through antenatal care services, and insecticides residual spraying (IRS), malaria incidence remains high in many regencies (Fig. 2)^8–11^. Moreover, few studies have been conducted to understand the vector drivers of transmission, i.e., characterizing malaria vector species, their ecology, and behaviors in Papua, Indonesia^12–15^. Therefore, a rapid entomological assessment was necessitated to obtain up-to-date data on vector transmission drivers to evaluate current methodologies and the potential of other new, and novel strategies in these eight regencies. This study aims to characterize focal and specific entomological endpoints to understand where and when transmission occurs.

## Methods

### Ethical statement

This study was approved by the Ethics Committee of Research in Health, Medical Faculty of Hasanuddin University, Makassar, Indonesia, Nos. 281/UN4.6.4.5.31/PP36/2019 and 40/UN4.6.4.5.31/PP36/2021.

### Site selection

Village mapping by API was the primary criteria informing site selection. Entomological assessments were conducted in eight regencies over a total of 150 days. Each regency included three Health Centres, and within each health centre catchment area, two villages were selected (a total of 48 villages). The sampling frame outlined depended on the site selection (based on higher transmission). This study was conducted in two separate periods. The first field activity was done in May–September 2019 in Keerom, Sarmi, Jayapura, Timika, and Boven Digoel Regencies. The second field activity was in the Yapen Islands, Waropen, and Asmat Regencies from November 2020–to February 2021. These study locations comprise 16 coastal areas, six riverine, 24 inlands, and two highlands. Entomological collections included adult and immature (larvae and pupae) sampling.

Based on 11 climate observation stations in 11 regencies in Papua Province, in 2019, the average temperature in Papua Province was 18.7–28.0 °C, with average air humidity 79.7–85.8%, maximum in May 93.0% and minimum 67.0% in September. The precipitation indicator showed a value between 1.562 and 5.413 mm^3^. Meanwhile, the average rainy days were 163–308 days^7^. Rainfall, temperature, and humidity (including seasonality) were not predictors of transmission—Fadilah *et. al.* demonstrated that levels of transmission intensity in Papua are highly heterogeneous, but they did not identify specific factors^16^. It is possible that heterogeneity in vector species distribution is a contributing factor, which this study aimed to characterize.

### *Anopheles* species analysis

All mosquitoes were identified taxonomically in the field laboratory using a dissecting microscope. The mosquito keys (J. Bonne-Webster et al*.*, 1953)^17^ were used for the identification of *Anopheles* mosquitoes, while the keys of Rozeboom and Knight 1946^18^ and Bryan 1974^19^ were used to distinguish species within the *Punctulatus* complex. The morphological identity of specimens (randomly selected from all sites and across all morphologically identified species) was molecularly identified using mosquito DNA barcoding towards the species verification^20,21^. Molecular confirmation of species was also performed on the collected *Anopheles* larval samples. The discrepancies between the results of adult *Anopheles* species (morphological versus molecular) identification methods were assessed using sensitivity as the percentage of true positives and positive predictive value (PPV) as the total number of true positives in the population.

### Entomological endpoints evaluated

Entomological evaluations included the feeding behaviour of vectors (where and when mosquitos bite humans, i.e., inside and outside human habitation), the human biting rate (HBR) (bites per person per night (bpn)), resting habits (indoor resting density), infectivity (sporozoite rate) and entomological inoculation rate (EIR) (infectious bites per person per night (ibpn)). The sporozoite rate was calculated as the number of mosquitoes infected with sporozoites divided by the number of mosquitoes examined. Meanwhile, EIR was calculated as the product of HBR and sporozoite rate. Adult mosquito collection was carried out using human landing catch (HLC) and indoor resting collections. Adult vector densities were assessed using standardized human-landing catch (HLC) methodologies^22^. Immature stage sampling of potential larval habitats^23^ was conducted daily during the study period. Adult sampling by regency allowed analysis of the spatio-temporal distribution of each species, vector species diversity, and abundance^22^.

### Mosquito collections

#### Human landing catch (HLC) collection

Adult mosquito collections were conducted through HLCs in four sentinel houses in each area^22^. Sentinel structures were chosen based on the structures being representative and residents being willing to participate in this study. HLCs were performed from 18.00 to 06.00 to evaluate *Anopheles’* indoor and outdoor landing densities in the houses of the selected villages using adult human volunteers who have provided written informed consent to participate (a total of eight persons, four persons indoors and four persons outdoors). Human biting rate (HBR calculated as bpn) was calculated from the total collected *Anopheles* using HLC per night per site divided by the number of volunteers. Moreover, in order to draw the periodicity of *Anopheles* biting behaviour, HBR was used and calculated as bite per person per hour (bph), the total collected *Anopheles* using HLC per hour per site divided by the number of volunteers. This periodicity chart was calculated using data only from the study site that was found to be related to *Anopheles* species.

#### Indoor resting collection

Adult mosquito collections were conducted through indoor resting collections (morning and night) using the manual aspirations method^24^. In the morning, collections were conducted in 10–20 randomly selected houses for 10–15 min per house. Night resting collections were performed every two hours from 18.00 to 06.00 in four houses without HLC activities. The indoor resting density (IRD) was calculated as the total *Anopheles* collected per sentinel house per night. The comparison of total mosquitos collected from indoor night resting collection versus mosquitos from HLCs was calculated by dividing the total number of night resting mosquitos by the total mosquitos taken from HLC.

#### Larval collection

Larval surveys were conducted on water bodies adjacent to human dwellings in the village or within the nearby forest following the guidelines described previously^22^. Larval collections were conducted in all water bodies that were potentially predicted to be larval habitats and categorized into eight habitat types of water body, namely pond/lake, ditch/gutter, seepage/spring/well, rain pool/puddles, stream margin, rice field, swamp and tidal depression. Quantification of *Anopheles* larval density (*D*) was calculated as the total number of *Anopheles* larvae divided by the total number of dips. Characteristic data groups compared with larval density included habitat types, habitat exposure (sunlight), habitat stability, flowing water speed, and vegetation^25–27^.

The location of water bodies in each study site was plotted on a map generated for each location. The potential risk of local malaria transmission was estimated based on the observed number of *Anopheles* larval sites on each map. Assuming that adult *Anopheles* can fly about 500 m from their oviposition or larval sites per night, possible areas of malaria transmission in the local map were drawn by plotting light red circles within a radius of 500 m. A higher risk area of malaria transmission may be visualized by a higher concentration of red color in the map.

All methods above were performed in accordance with the relevant guidelines and regulations^22^.

### Mosquitoes sample preparation and DNA isolation

#### Sample preparation

*Anopheles* samples were dissected into two parts: head and thorax for *Plasmodium* (a proxy for sporozoite) detection, and other parts were used for species confirmation by molecular tools. Ovaries were dissected to determine parity (parous and nulliparous). Collected *Anopheles* specimens were desiccated individually in 1.5 ml Eppendorf microtubes with silica gel and stored at 4ºC until analysis. The head and thorax of the mosquito were dissected and transferred into a 1.5 ml microtube. The head and thorax were homogenized with Teflon pestles in 100 µl water. A Chelex extraction protocol was used for the DNA isolation process^28^. DNA was used immediately for a polymerase chain reaction (PCR) or stored at − 20 °C for later analysis.

### DNA amplification

#### *Plasmodium* sporozoite detection

A set of primers COX-IF (5′ AGAACGAACGCTTTTAACGCCTG 3′) and COX-IR (3′ ACTTAATGGTGGATATAAAGTCCATCCwGT 5′), to amplify a polymorphic fragment in the COX-I gene^29^, and two Plasmodium-specific primers based on the sequence of the small subunit ribosomal RNA (ssrRNA), namely rPLU (5 and 6) and rFAL (1 and 2); rVIV (1 and 2)^30^ were used for plasmodium sporozoite detection.

#### Species confirmation

Molecular species confirmation was performed using ribosomal DNA internal transcribed spacer region two and mitochondrial DNA cytochrome oxidase subunit one locus were previously described^31^.

## Data analysis

Data collection was designed using a tablet-based survey platform based on the Open Data Kit system (ODK)^32^. ODK Collect was selected as a frontend open-source Android application, and ODK Aggregate was used as linked to the custom-build database. Quantitative data was managed and analyzed using Microsoft Office Excel essential functions and an open-source software, Rstudio version 2023.03.0 + 386 on R version 4.1.0^33,34^. A one-way analysis of variance (ANOVA) was used to compare variations in HBR of mosquitos in genera and *Anopheles* species collected from different geographical landscapes, as well as in larval density of *Anopheles* among the habitat types and conditions. If the p-value was less than the significance level (0.05), there were significant differences between the groups being compared. The Tukey Honest Significant Differences (Tukey HSD) test was used to check for all possible pairs of all HBRs and larval densities. Moreover, the relationship between *Anopheles* HBR and larval habitat productivity in each study location was tested using multiple regression analysis. Here, larva habitat productivity was defined as the average larval density and percentage of *Anopheles* positive index habitat.

The taxonomic relationships between molecularly characterized samples were inferred using the Maximum Likelihood method and the Tamura-Nei model^35^. Initial tree(s) for the heuristic search were obtained automatically by applying Neighbor-Join and BioNJ algorithms to a matrix of pairwise distances estimated using the Maximum Composite Likelihood (MCL) approach and selecting the topology with superior log likelihood value. The tree was drawn to scale, with branch lengths measured in the number of substitutions per study site. Evolutionary analyses were conducted in MEGA X^36^.

## Results

### Adult mosquito collections

**Figure 3:**
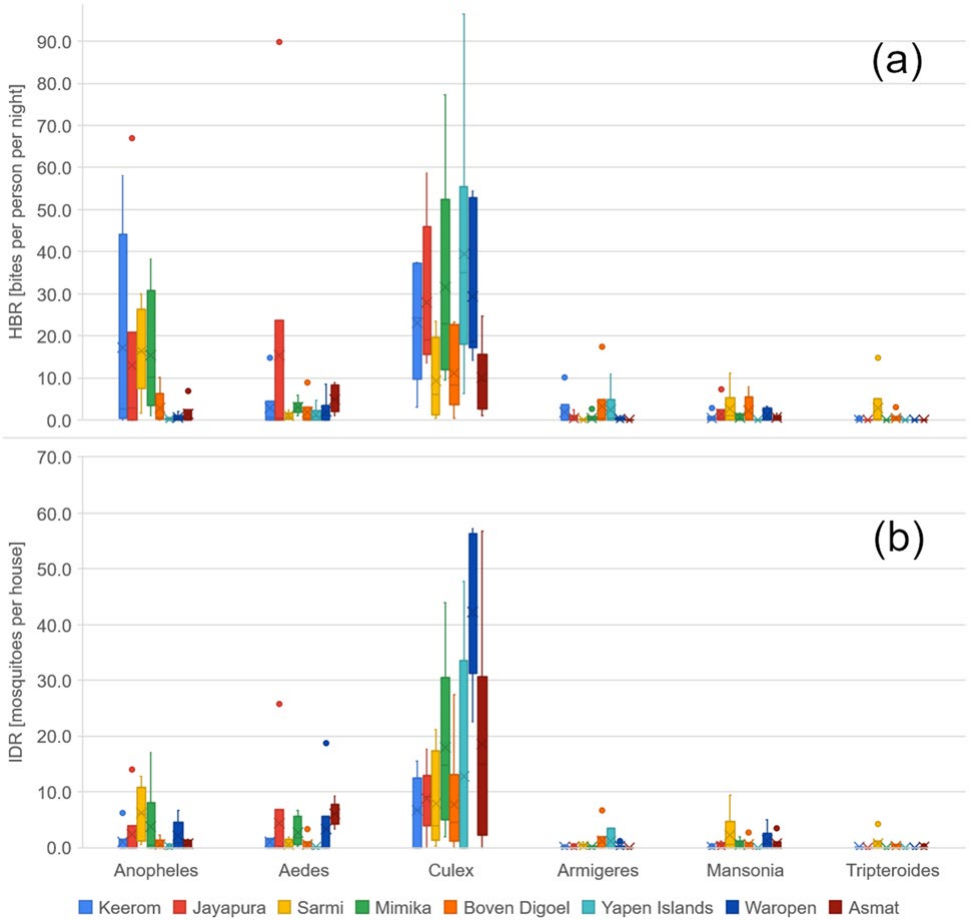
Morphologically identified adult mosquitos were collected during (**a**) HLC (in HBR) and (**b**) indoor night resting collection (in IRD) in eight regencies.

Of the 19,478 mosquito specimens, 14,486 (74.4%) were collected from HLCs, and 4992 (25.6%) were collected from indoor resting collections and were dissected during the 2019 to 2021 study periods. Eleven genera of mosquitoes were identified morphologically. Figure 3 shows the average of adult mosquitos collected during HLC (written in HBR (bpn)) and indoor night resting collection (written in IRD) in eight regencies (Supplementary File 1). Most specimens collected belonged to the following six genera—*Anopheles*, *Aedes, Culex, Armigeres, Mansonia* and *Tripteroides*. Other samples included *Coquilletidia, Ficalbia, Lutzia, Malaya, Toxorhychites,* and *Uranotaenia*. Approximately 479 samples could not be identified to their genus due to being physically destroyed or exposed to the fungus. Over 75% of non-*Anopheles* mosquitos were *Culex*, including *Culex quinquefasciatus, Cx. gellidus, Cx. tritaeniorhyncus, Cx. sinensis, Cx. pseudovisnhui, Cx. mimulus, Cx. sitiens, Cx. visnhui, Cx. papuensis, Cx. halifaxi* and *Cx. bitaeniorhynchus*. *Culex* was found in large numbers in all study sites. Moreover, 2,034 mosquitoes (12.8% of the total non-Anopheles) identified as *Aedes* were caught during the study. The sub-district with the most *Aedes* came from Namblong, Jayapura Regency. The species found included *Ae. aegypti*, *Ae. kochi*, *Ae. albopictus*, *Ae. finlaya*, *Ae. vexans*, *Ae. poecilius* and *Ae. mucidus*. *Mansonia* was primarily found in Sarmi Regency, especially in the Tor Atas Sub-district. The *Mansonia* species found in this study were *Mansonia uniformis*, *Ma. papaensis*, *Ma. bonneae* and *Ma. indiana*.

Though adult *Anopheles* were collected from all regencies, they were not necessarily captured in all study sites. Human biting rates (bpn) generated from HLCs across the study site varied from 0 to 66.9 bpn with an average of 8.4 ± 15.3 bpn. Meanwhile, indoor night resting collections demonstrated a capture rate from 0 to 39.0 *Anopheles* per sentinel house per night, with an average of 2.1 ± 4.0. Ten *Anopheles* species were morphologically identified, with more than 95% of samples belonging to the Punctulatus Group, which includes *An. koliensis*, *An. punctulatus*, *An. farauti* and *An. hinesorum*.

The variation of HBRs by mosquito genera and *Anopheles* species were mapped to the collection site (eight regencies) and the geographical landscape of study sites, i.e. coastal, highland, inland, and riverine areas. No significant differences were found in the variation of HBRs with study sites and geographical landscape, except for *An. farauti* that had a significant correlation with the study site (*p-value* = 9.42 × 10^–7^), where further Tukey HSD test showed a significant value for Mimika Regency.

**Figure 4:**
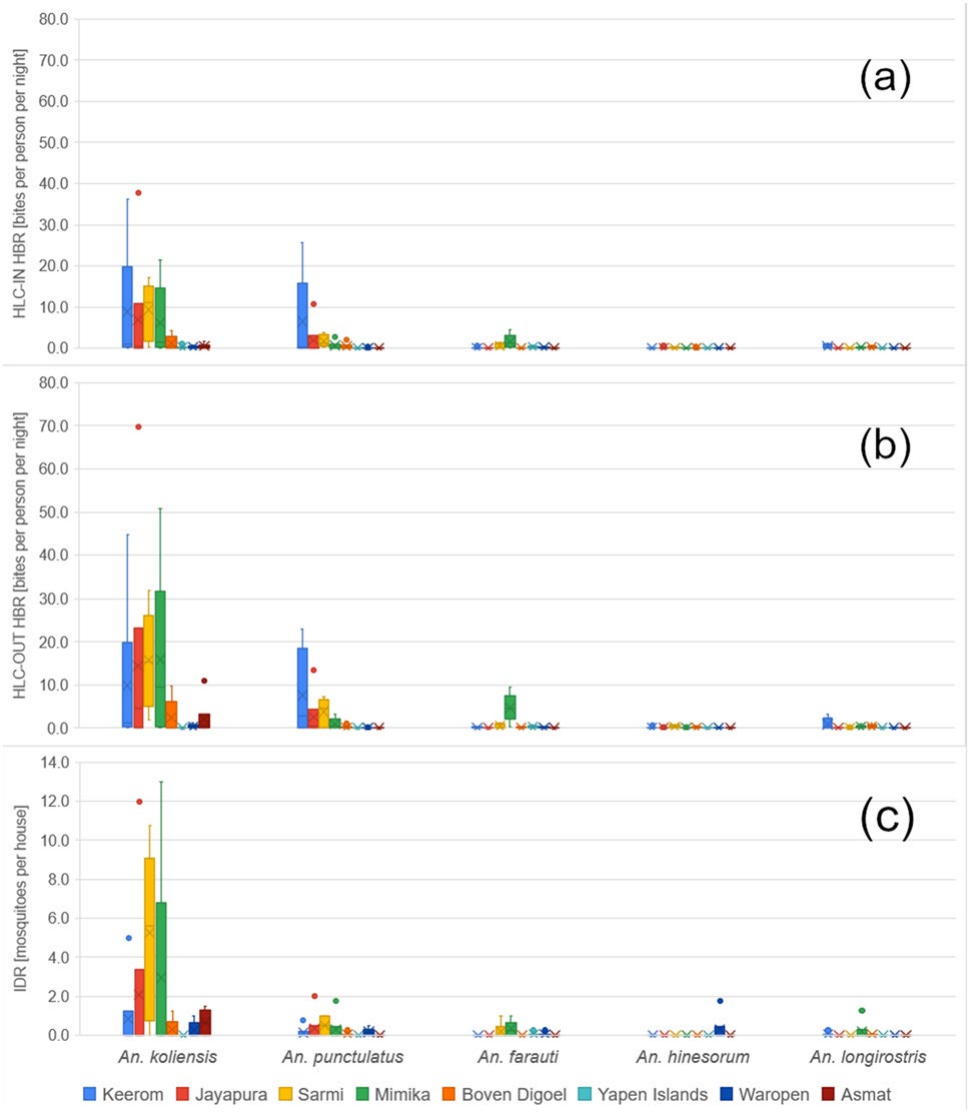
Morphologically identified five most abundant adult *Anopheles* species collected during HLC (in (**a**) indoor HBR and (**b**) outdoor HBR) and (**c**) indoor night resting collection (in IRD).

The 29 samples from the Hyrcanus Group included *An. nigerrimus*, *An. nitidus* and *An. peditaeniatus* and were found mainly in Waropen Regency. *An. longirostris* was collected in five regencies. Other species consisted of three *An. kochi* and one *An. vagus* samples which were found in Waropen regency. Only a small number of *Anopheles* were captured in the Yapen Islands. Figure 4 shows box and whisker charts of the five most commonly collected *Anopheles* during HLCs and indoor night resting collection in the eight regencies. Overall, in the eight regencies outdoors biting was approximately twice as much indoors, with the average HBR from indoor HLCs HBR being 6.1 ± 12.3 bpn and that from outdoor HLCs HBR being 10.8 ± 19.1 bpn. Approximately 70% of the specimens captured indoors and outdoors consisted of *An. koliensis*. Although *An. punctulatus* demonstrated a preference for outdoor biting, there was a higher proportion of this species found indoors when compared to *An. koliensis*. The average IRD from indoor night resting collections of *An. koliensis* and *An. farauti* was less than half of their indoor HBR, meanwhile the average IRD of *An. punctulatus* was slightly more than half of its indoor HBR.

Table 1 depicts the five most common *Anopheles* species present based on land type of study site locations. *An. koliensis* was found in large numbers in all types of areas. *An. punctulatus* and *An. hinesorum* were not found in the Yapen Islands and Asmat regencies, where these sites are mostly coastal and riverine areas. Otherwise, *An. farauti* was found proportionally in large numbers in the coastal areas of Mimika, Sarmi, and Yapen Islands, and less common in Keerom and Boven Digoel and not in Jayapura and Asmat. *An. longirostris* was mostly found in the inland areas.

**Table 1:** Morphologically identified average of five most abundant adult *Anopheles* species collected during HLC (in HBR) and indoor night resting collection (in IRD) divided by land type area.

Regency	Punctulatus Group* (*Punctulatus* complex)*												*An. longirostris*		
	*An. koliensis*			*An. punctulatus*			*An. farauti*			*An. hinesorum*					
	HLC-IN*^1^	HLC-OUT*^2^	Resting*^3^	HLC-IN	HLC-OUT	Resting	HLC-IN	HLC-OUT	Resting	HLC-IN	HLC-OUT	Resting	HLC-IN	HLC-OUT	Resting
	HBR [bpn]*^4^	HBR [bpn]	IRD*^5^	HBR [bpn]	HBR [bpn]	IRD	HBR [bpn]	HBR [bpn]	IRD	HBR [bpn]	HBR [bpn]	IRD	HBR [bpn]	HBR [bpn]	IRD
Coastal	2.9 ± 5.8 (186)*^6^	5.4 ± 10.3 (345)	1.8 ± 3.7 (112)	0.5 ± 1.2 (34)	1.1 ± 2.3 (70)	0.2 ± 0.4 (11)	0.7 ± 1.2 (45)	1.2 ± 2.6 (75)	0.1 ± 0.3 (9)	0.0 ± 0.1 (2)	0.1 ± 0.3 (8)	0.1 ± 0.4 (7)	0.0 ± 0.0 (0)	0.0 ± 0.0 (0)	0.0 ± 0.0 (0)
Highland	0.6 ± 0.9 (5)	0.8 ± 1.1 (6)	0.0 ± 0.0 (0)	0.0 ± 0.0 (0)	.0 ± .0 (0)	0.0 ± 0.0 (0)	0.0 ± 0.0 (0)	0.0 ± 0.0 (0)	0.0 ± 0.0 (0)	0.0 ± 0.0 (0)	0.0 ± 0.0 (0)	0.0 ± 0.0 (0)	0.1 ± 0.2 (1)	0.0 ± 0.0 (0)	0.0 ± 0.0 (0)
Inland	5.8 ± 10.9 (561)	10.8 ± 18.6 (1037)	2.4 ± 4.4 (226)	1.9 ± 4.1 (183)	2.6 ± 4.8 (254)	1.2 ± 4.7 (117)	0.1 ± 0.3 (13)	0.6 ± 1.6 (61)	0.1 ± 0.2 (8)	0.1 ± 0.1 (5)	0.1 ± 0.2 (9)	0.0 ± 0.0 (0)	0.2 ± 0.4 (16)	0.4 ± 0.8 (37)	0.1 ± 0.3 (7)
Riverine	0.5 ± 0.7 (11)	2.1 ± 4.4 (51)	0.7 ± 0.7 (16)	0.0 ± 0.0 (0)	0.0 ± 0.0 (0)	0.0 ± 0.0 (0)	0.0 ± 0.0 (0)	0.0 ± 0.0 (0)	0.0 ± 0.0 (0)	0.0 ± 0.0 (0)	0.0 ± 0.0 (0)	0.0 ± 0.0 (0)	0.0 ± 0.0 (0)	0.0 ± 0.0 (0)	0.0 ± 0.0 (0)
Total	4.0 ± 8.5 (763)	7.5 ± 14.8 (1439)	1.8 ± 3.8 (354)	1.1 ± 3.0 (217)	1.7 ± 3.7 (324)	0.7 ± 3.4 (128)	0.3 ± 0.8 (58)	0.7 ± 1.9 (136)	0.1 ± 0.2 (17)	0.0 ± 0.1 (7)	0.1 ± 0.2 (17)	0.0 ± 0.3 (7)	0.1 ± 0.3 (17)	0.2 ± 0.6 (37)	0.0 ± 0.2 (7)

*^1^Indoor human landing catch, *^2^Outdoor human landing catch, *^3^Indoor night resting collection, *^4^Human biting rate [bite per person per night], *^5^Indoor resting density, *^6^Number in brackets is the total number of mosquitos.

**Figure 5:**
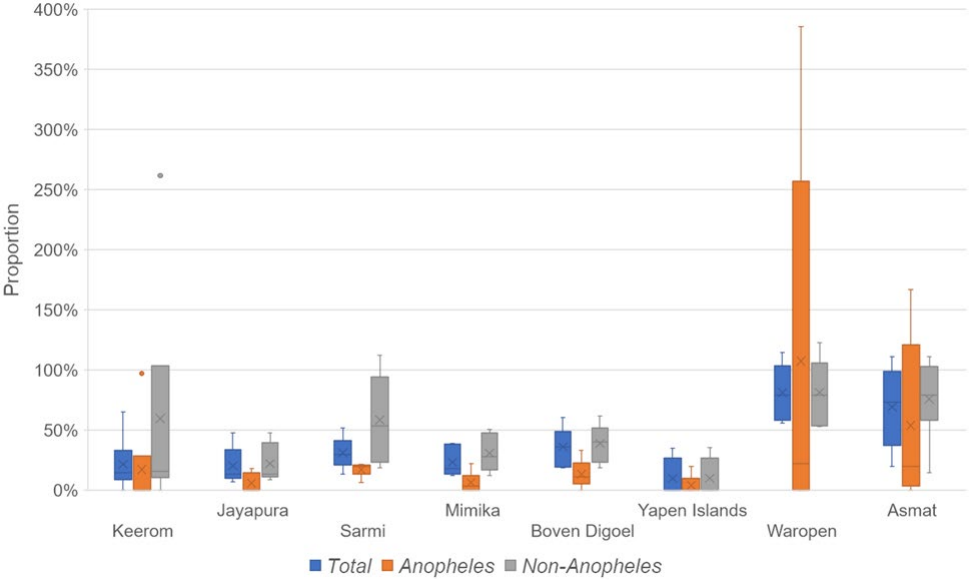
The proportion of overall Anopheles and non-Anopheles mosquitos was collected between indoor night resting versus HLC methods in each study site.

A total of 560 (15.4%) of the 3646 *Anopheles* collected were from indoor night resting collections, and only two *Anopheles* were collected from morning resting in the eight regencies. Figure 5 shows box and whisker charts of the proportion of overall *Anopheles* and non-*Anopheles* mosquitos collected between indoor night resting versus HLC methods in each study site. Except for Waropen and Asmat regencies, more *Anopheles* were captured in indoor HLCs than in the indoor night resting collections.

The proportions of non-*Anopheles* collected from indoor night resting were higher than that of *Anopheles*. Data from Waropen and Asmat regencies demonstrated that a significant number of *Anopheles* (from half that of HLC collections to several times higher) were found in indoor night resting collections. Data demonstrates that in all study sites, more non-*Anopheles* rested indoors than *Anopheles*.

### Vector incrimination result

From a total of 3458 *Anopheles* collected from the five regencies (Keerom, Jayapura, Sarmi, Mimika, and Boven Digul), only 18 mosquitoes from five villages were found to carry *Plasmodium* (Table 2). A total of 12 positive *Anopheles* mosquitoes were collected from Skofro, a village in Keerom regency, where the second highest number of *Anopheles* were collected (total: 489 *Anopheles*, HBR = 58.0 bpn and IDR = 6.25 per house per night). Based on molecular *Anopheles* species confirmation, the positive mosquitoes included 16 *An. koliensis* and two *An. punctulatus*. Moreover, 15 of these positive mosquitoes were collected with HLCs (eight from HLC outdoors and seven from HLC indoors) and the remaining three were from indoor night resting collections. Calculated Plasmodium positivity rates of each study site demonstrated that Karya Bumi was the highest at 4.35%, followed by Skofro, which was the next highest at 2.67%. The *Plasmodium* positivity rate from the five regencies calculated was 0.58%. The EIR for these five villages demonstrated that Skofro village had the highest transmission risk (EIR = 1.55 ibpn).

**Table 2:** Detection of Plasmodium among the *Anopheles* collected from five regencies.

Regency	District	Village	*Anopheles* species (molecular)	Positive plasmodial DNA		Uninfected	Sporozoite rate (%)	HBR*^2^ (bpn)	EIR*^3 (ibpn)^
				Pf*^1^	*Plasmodium sp.*				
Keerom	Arso Timur	Skofro	*An. koliensis*	0	6*^4O^, 5*^4I^	438	2.67	58.00	1.55
			*An. punctulatus*	0	1*^4I^				
		Pikere	*An. koliensis*	1*^5^	1*^5^	297	1.00	20.13	0.20
			*An. punctulatus*	0	1*^4O^				
Jayapura	Namblong	Karya Bumi	*An. koliensis*	0	1*^4O^	22	4.35	4.25	0.18
		Hanggey Among	*An. koliensis*	0	1*^4I^	555	0.18	66.88	0.12
Sarmi	Pantai Barat	Webrau	*An. koliensis*	0	1*^5^	203	0.49	25.00	0.12

*^1^ *Plasmodium falciparum,* *^2^ Human biting rate (bite per person per night), *^3^ Entomological inoculation rate, *^4^ Collected from HLC, ^I^ indoor and ^O^ outdoor, *^5^ Collected from night indoor resting.

### Species identification using barcoding ITS2

Of the 551 molecularly examined samples randomly selected from different study sites, the nine species identified included *An. koliensis*, *An. punctulatus*, *An. farauti*, *An. hinesorum*, *An. longirostris*, *An. peditaeniatus*, *An. tesselatus, An. vagus* and *An. kochi*. *Anopheles* samples that previously were not identified as species (n = 47) were also included in the molecular analysis, and, as a result, a total of 534 samples were confirmed to be species level (17 could not be identified). Table 3 shows the comparison between morphological and molecular species identification. Sensitivity showed that only four species had scores of more than 75%—*An. koliensis*, *An. longirostris*, *An. kochi* and *An. vagus*, where the last two species only found a single mosquito each. The discrepancies between species identification results mostly occurred in the same sub-species groups, as shown in Table 5. Moreover, four samples of *An. tesselatus* that were not identified morphologically were identified molecularly. Positive predictive value (PPV) showed two species previously identified as *An. nigerimus* and *An. nitidus* from Waropen regency were re-identified as *An. peditaenitus*, which also belongs to the Hyrcanus Group. PPV of *An. punctulatus* (1.33%) showed that only a single true positive was identified from a total predicted 75 mosquitoes from morphological identification.

**Table 3:**
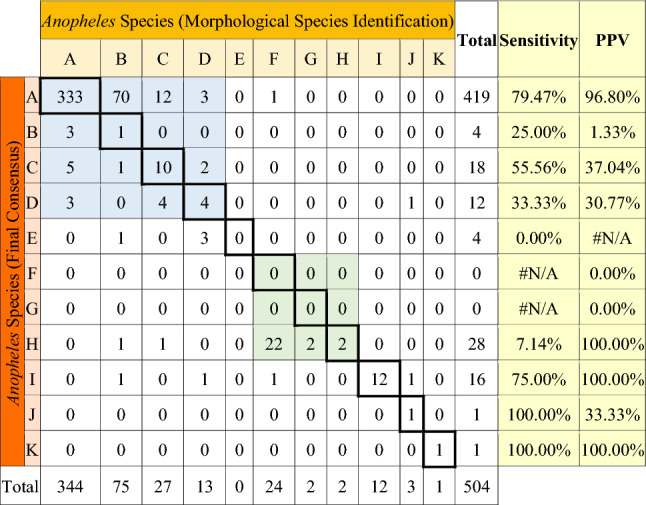
Morphological vs. molecular species identification of *Anopheles*.

Blue shading points to the Punctulatus Group and green shading the Hyrcanus Group (A: *An. koliensis,* B: *An. punctulatus,* C: *An. farauti,* D: *An. hinesorum,* E: *An. tesellatus,* F: *An. nigerimus,* G: *An. nitidus,* H: *An. peditaenitus,* I: *An. longirostris,* J: *An. kochi,* K: *An. vagus,* PPV: Positive Predictive Value).

### *Anopheles* biting behavior

**Figure 6:**
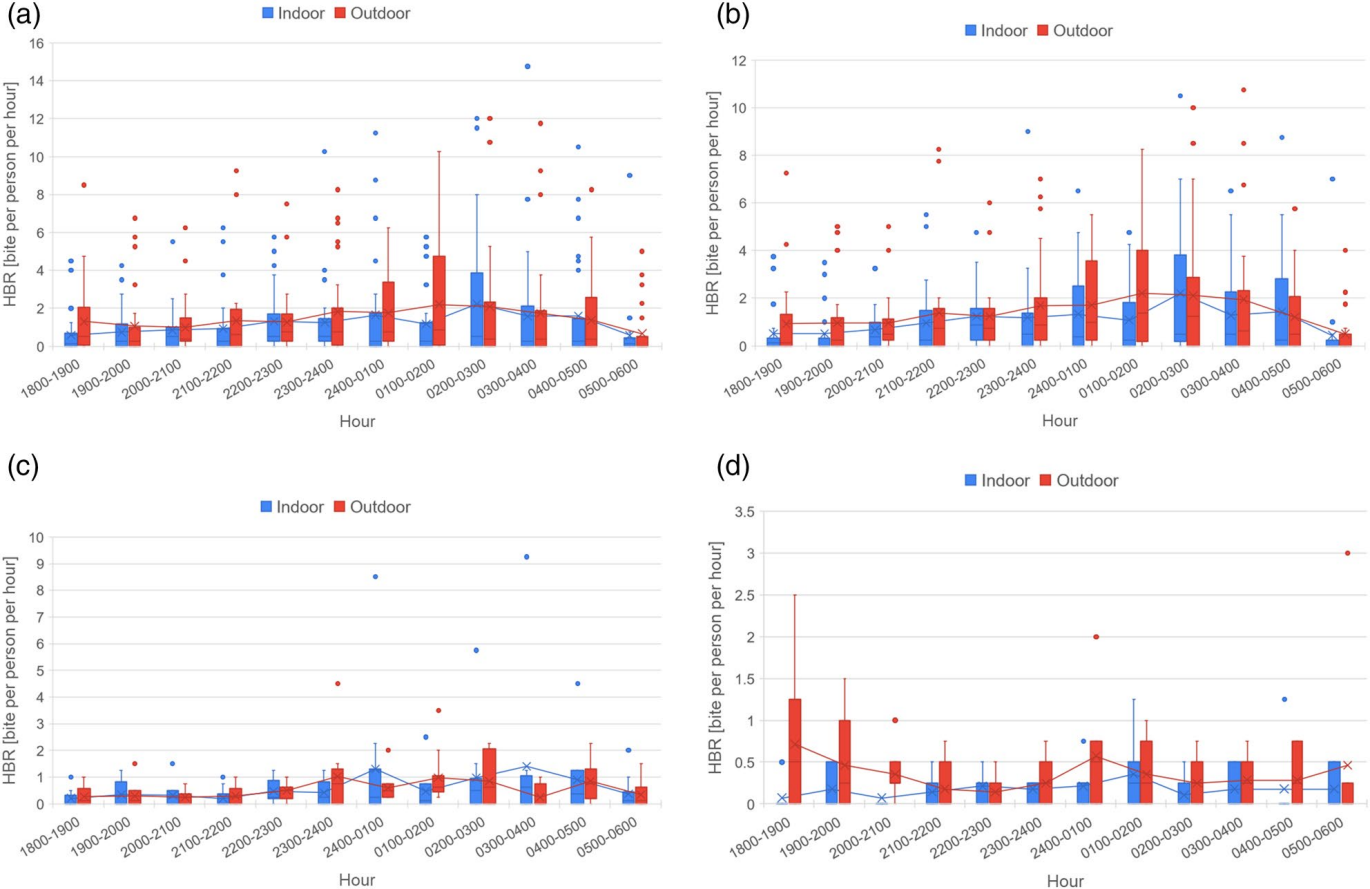
Periodicity of *Anopheles* human biting rate in bite per person per hour (bph). (**a**–**d**) presented the overall periodicity of human biting rate (data plotted from 28 study sites), *An. koliensis* (22 study sites), *An. punctulatus* (10 study sites), and *An. farauti* (seven study sites), respectively.

Figure 6 shows the periodicity charts of *Anopheles* biting behaviour as HBR (bph) of overall *Anopheles* and the three most collected species i.e., *An. koliensis*, *An. punctulatus* and *An. farauti*. Figure 6a depicts the overall *Anopheles* HBR, demonstrating that *Anopheles* bites from the beginning of the collection time in 18.00 and continues through the night. A peak in periodicity was seen at 01.00 to 02.00 h after midnight. Overall hourly HBR indoor = 1.2 ± 2.2 bph varied from 0 to 14.8 bph, meanwhile hourly HBR outdoor = 1.5 ± 2.3 bph varied from 0 to 12.0 bph. Here, as to *Anopheles* biting periodicity, there were no significant differences between hourly indoor and outdoor HBR periodicities. The HBR of *An. koliensis* (Fig. 6b) and *An. punctulatus* (Fig. 6c), as the major mosquitoes collected had similar time of late biting profiles. Meanwhile in contrast, *An. farauti* (Fig. 6d), which, was mostly collected from the coastal area of Mimika Regency had a different curve; where the host-seeking was steady throughout the night but peaking in the early hour (18.00–19.00). Each species' respective hourly HBR indoor and HBR outdoor were *An. koliensis* 1.1 ± 1.8 bph (varied from 0 to 10.5 bph) and 1.4 ± 2.0 bph (0–10.8 bph), *An. punctulatus* 0.6 ± 1.3 bph (0–9.3 bph) and 0.5 ± 0.7 bph (0–4.5 bph), *An. farauti* 0.2 ± 0.3 bph (0–1.3 bph) and 0.4 ± 0.5 bph (0–3.0 bph).

### Larval site surveys

This study observed water bodies' surveillance in eight regencies, as presented in Table 4. The most common habitat type was animal hoof-prints, or vehicle tire tracks made rain pool/puddles that included puddles, rainwater containers within residential areas, and water in old tires or other discarded items around houses. Furthermore, ditches or gutters around the houses or along the roadside in front of the houses were found with larvae. Man-made ponds were found in the yard close to houses and contained larvae. These larval habitats were characterized and present less than 100 m from the nearest residential structure. Rice field-based habitats were only observed in Jayapura, and tidal depression sites were found in coastal areas like Sarmi and Yapen Islands.

**Table 4:** Percentage of *Anopheles* larvae’s habitat index in eight regencies.

Habitat type	Keerom	Jayapura	Sarmi	Mimika	Boven Digoel	Yapen Islands	Waropen	Asmat	Total
Pond/lake	40.4% (47)*	27.3% (33)	54.9% (51)	38.9% (54)	28.1% (96)	50.0% (54)	46.0% (87)	36.4% (33)	40.2% (455)
Ditch/gutter	28.4% (74)	37.7% (53)	61.9% (42)	23.9% (71)	31.4% (51)	0.0% (10)	32.5% (160)	9.8% (41)	31.1% (502)
Seepage/spring/well	28.6% (7)	33.3% (18)	27.3% (11)	–	21.6% (37)	66.7% (6)	37.5% (8)	15.6% (32)	26.1% (119)
Rain pool/puddles	22.2% (27)	26.1% (69)	25.4% (59)	27.9% (68)	15.3% (111)	23.4% (64)	62.2% (201)	12.2% (74)	33.3% (673)
Stream margin	28.6% (7)	50.0% (2)	60.0% (15)	0.0% (3)	0.0% (11)	0.0% (1)	20.0% (5)	0.0% (1)	28.9% (45)
Rice field	–	50.0% (2)	–	–	–	–	–	–	50.0% (2)
Swamp	0.0% (2)	25.0% (4)	0.0% (3)	0.0% (10)	41.2% (17)	25.8% (31)	80.0% (10)	–	31.2% (77)
Tidal depression	–	–	0.0% (2)	–	–	0.0% (1)	–	–	0.0% (3)
Total	30.5% (164)	30.9% (181)	44.3% (183)	27.7% (206)	23.2% (323)	32.3% (167)	48.6% (471)	16.6% (181)	33.7% (1876)

*Numbers in the brackets are the total number of water bodies.

**Figure 7:**
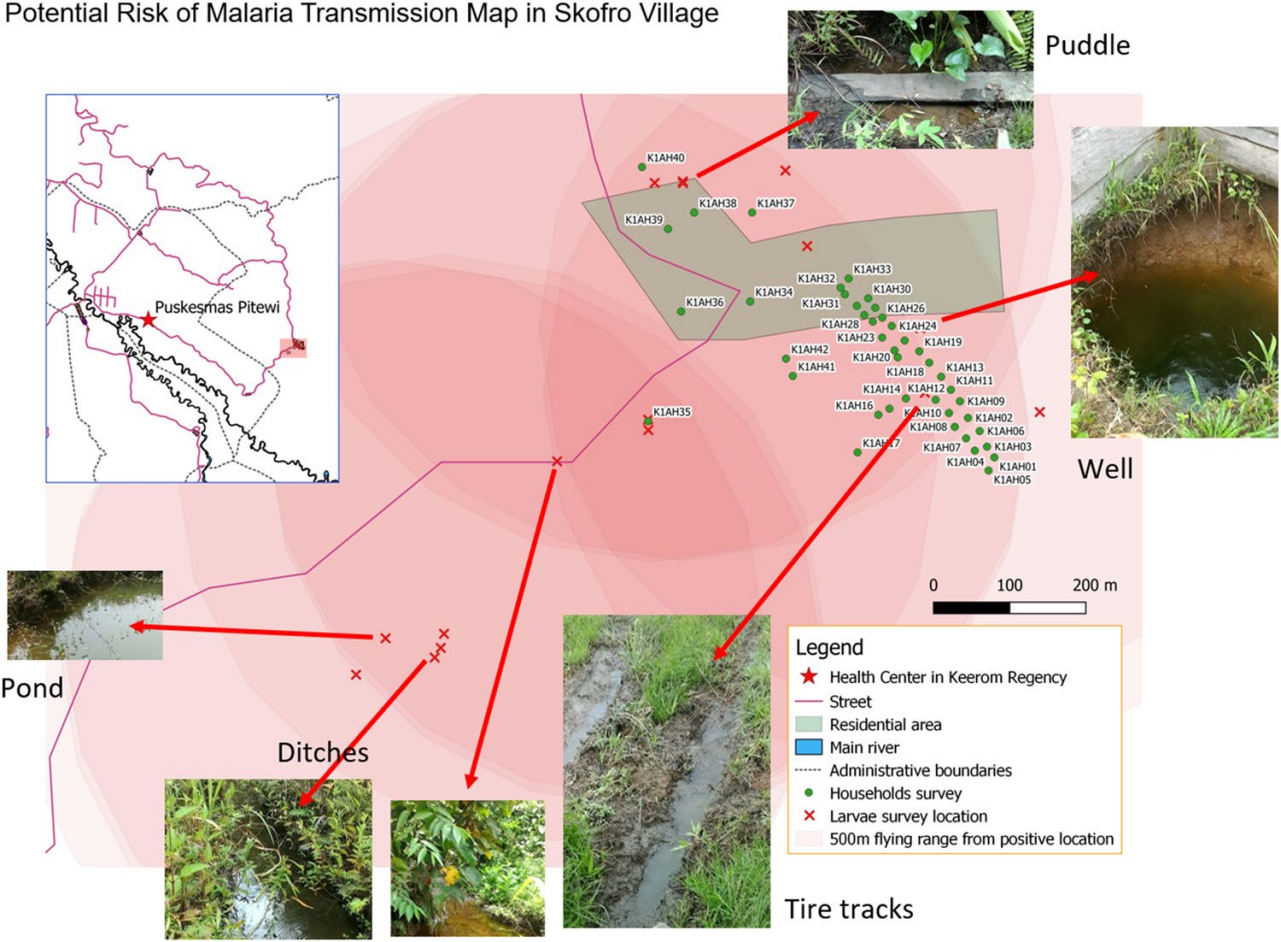
Map of the potential risk of malaria transmission in Skofro Village, Keerom Regency.

A total of 1876 water bodies consisting of 1076 human-made and 800 natural sites were observed. Of these, 632 (33.7%) water bodies were positive for *Anopheles* larvae. The presence or absence of data was unclear or incomplete from 242 (12.9%) water bodies. Overall, the habitat positivity index for *Anopheles* larvae was 33.7%, varying from 16.6% in Asmat to 48.6% in Waropen (Table 4). Approximately 93.67% ± 3.89% *Anopheles* positive water bodies were located in residential areas or near houses. Approximately 31.63% ± 18.94% of water bodies were permanent, 44.78% ± 20.04% were semi-permanent, and 23.37% ± 12.69% were temporary. Permanent and semi-permanent water bodies found in the surrounding residential areas that responded to maintaining highly productive *Anopheles* breeding sites were mostly ponds, ditches/gutters, and wells, while temporary water bodies consisted of rain pools or puddles (Table 4). Data analysis from larval density showed that no significant differences in habitat types (*p*-value = 0.999) and habitat conditions were found, namely habitat stability (*p*-value = 0.199), flowing water ripples (*p*-value = 0.124), and the presence of vegetation (*p*-value = 0.8). Meanwhile, a positive relationship was found for the correlation with habitat exposure, where further Tukey HSD test showed a significant difference between sunlight and shaded habitat (*p*-value = 0.008). The multiple regression analysis between *Anopheles* HBR and the larval habitat productivity, which is defined as the average of *Anopheles* larval density and percentage of *Anopheles* positive index habitat, did not show any significant positive relationship with the larval density (*p*-value = 0.921) but showed slightly positive relationship with the percentage of positive index habitat (*p*-value = 0.0463). *Anopheles*-positive water bodies and their habitat types were plotted along with the estimated potential risk of local malaria transmission in Fig. 7.

### Evolutionary analysis

**Figure 8:**
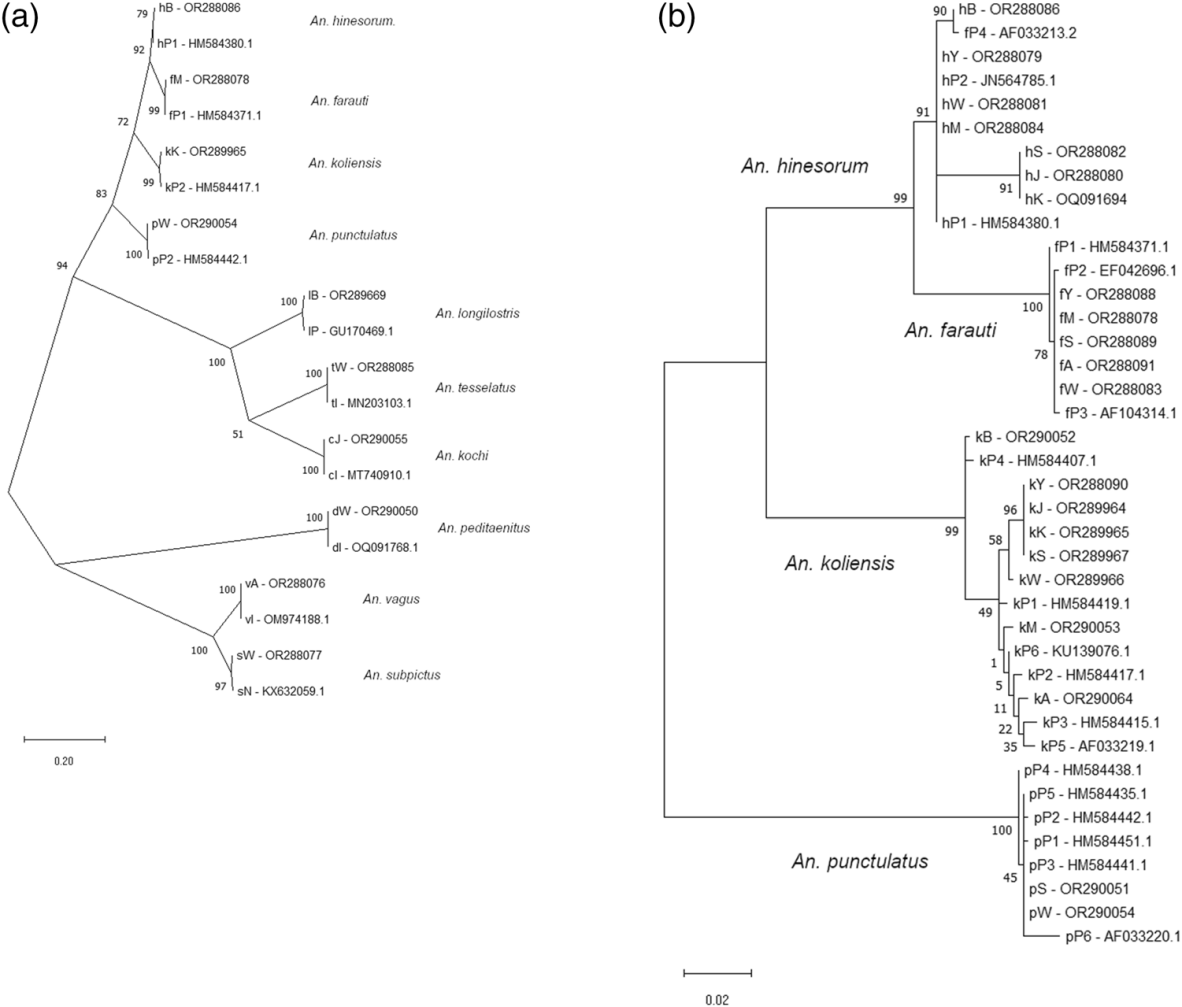
A phylogenetic tree depicts the evolutionary relationship between *Anopheles* samples from each study site based on ITS2 analysis. (**a**) Presented the relationship between represented samples of each species, and (**b**) for Punctulatus group samples from each study site—detailed notation sample codes presented in Supplementary File 2.

The findings from entomological assessment in eight regencies are summarised in Table 5. ITS2 data from adults and larvae (number of samples analyzed versus total samples caught = 10%) was used to generate taxonomic relationships. A total of 10 *Anopheles* species were identified, namely *Anopheles koliensis*, *An. punctulatus*, *An. farauti*, *An. hinesorum*, *An. longirostris*, *An. peditaeniatus*, *An. tesselatus, An. vagus, An. subpictus* and *An. kochi*. Except *An. subpictus,* all adult *Anopheles* of each species was confirmed molecularly. *An. subpictus* was identified only during from its larval sampling. The evolutionary relationship between represented samples of each species (20 nucleotide sequences) was analyzed using gen ITS2 with the tree’s highest log likelihood (− 5779.93) presented (Fig. 8a). Meanwhile, Fig. 8b presents the phylogeny all species of Puntulatus Group samples collected from each study site compared to different areas (mostly samples taken from Papua New Guinea). The percentage of trees in which the associated taxa clustered was shown next to the branches. All samples used in the phylogeny tree analysis are listed in Supplementary File 2.

**Table 5:** Summary of *Anopheles* species distribution in eight regencies.

	Keerom	Jayapura	Sarmi	Mimika	Boven Digoel	Yapen Islands	Waropen	Asmat
*An. koliensis*	M, R, L, +	M, R, L, +	M, R, L, +	M, R, L	M, R, L	M	M, R, L	M, R, L
*An. punctulatus*	R, +	R	R, L	–	M, R	–	M, R	–
*An. farauti*	–	M	M, R	M, R	M	M, R, L	M, R, L	M, L
*An. hinesorum*	M, R	L	L	R	M, L	M, L	M, R, L	–
*An. tesellatus*	–	–	–	–	–	–	M	–
*An. longilostris*	M, R	–	–	M	M, R	–	M, R	–
*An. peditaeniatus*	–	–	–	M, L	–	–	M, R	–
*An. kochi*	–	L	–	–	–	–	M, R, L	–
*An. subpictus*	–	–	–	–	–	–	L	–
*An. vagus*	–	–	–	–	–	–	R, L	L

M: Adult *Anopheles* collected during HLC, R: Adult *Anopheles* collected during indoor resting, L: *Anopheles* larvae collection, + : *Anopheles* positive carried plasmodial DNA.

## Discussion

The Punctulatus Group, identified as a potential major vector in this study, is commonly found in the Australasian region^37^ and contains malaria and lymphatic filariasis vectors. This group is distributed from Maluku, Papua, Papua New Guinea, northern Australia, the Solomon Islands, and Vanuatu. Members of the Punctulatus Group have a variety of habitats, ranging from coastal areas to inland areas. *An. farauti 1,* generally found within a kilometer from the coast, was documented in Mimika, Sarmi regencies and also from Yapen, Waropen, and Asmat regencies, while *An. hinesorum* (*An.farauti 2*) and *An. koliensis* were generally found inland^13^. During this study, the presence of *An. hinesorum* was observed in seven of eight regencies (except Asmat) (Fig. 8). *Anopheles* species from the Punctulatus Group from Papua have high morphological similarity (Table 3), and with a low PPV result for *An. punctulatus* (1.33%) showing some unusual morphological characteristics in *An. koliensis* that might be similar to *An. punctulatus* based on the mosquito keys used^38,39^. Meanwhile, the members of the *An. farauti* complex is morphologically indistinguishable and can only be typed to species using molecular analysis^12^. The phylogenetic construct based on the ITS2 gene revealed that the Punctulatus Group collected from eight regencies in Papua are monophyletic. Since regencies included in this study possessed variety of land types and the sampling sites did not represent all of them, a more comprehensive sampling strategy across all land types in a regency would enable a better characterization of species diversity.

Malaria vector control in Papua has been based on distributing indoor functioning ITNs and indoor residual spraying (IRS)^40^. This study found approximately 2.6 ITNs per house, with IRS coverage being incomplete (only 28.4% of the households in the study sites), including several sites where the IRS was never implemented (unpublished data). The effectiveness of these interventions relies on several factors including susceptibility of mosquitoes to the insecticides used, adequate coverage, quality and timely implementation, user acceptance or compliance, and especially vector behaviours^41–43^. While factors that can limit the effectiveness of existing interventions are extremely important and must be addressed, even full implementation of core interventions may not halt malaria parasite transmission across all settings^44^. Both intervention methods (ITNs and LLINs) mainly impact anthropophilic mosquitoes that are endophagic and endophilic (respectively), enabling a proportion of vectors to escape from contact with insecticide-treated surfaces and maintain a certain level of transmission^10,11^.

The findings from this cross-sectional study, which included 48 different locations in Papua’s eight regencies, demonstrated that Punctulatus Group mosquitoes were present in all regencies. Previous studies employing longitudinal observations documented different results in terms of abundance and behaviour, pointing to possible changes over time or differences in data based on sampling frameworks^15^.

Only 18 (0.52%) of the 3458 *Anopheles* females evaluated were found to carry *Plasmodium* DNA. This is likely an underrepresentation of infected mosquitoes, as previous studies demonstrate that these vectors play a major role in malaria transmission in Papua^12,13,15,45^. Larval sites found in residential areas and near house dwellings also support their role in transmission. Overall *An. koliensis*, *An. punctulatus* and *An. farauti* were found to bite from dusk at 18.00 until dawn at 06.00 and had a peak at 01.00–02.00 h after midnight (Fig. 6a), with 40% of biting happening indoors—pointing to the open structure of houses and opportunistic *Anopheles* feeding patterns. This biting peak primarily reflected the behavior of the largest contributor to biting densities—*An. koliensis* (Fig. 6b). The biting peak of *An. punctulatus* demonstrated a slight difference between 02.00 and 03.00 h (Fig. 6c). Meanwhile in contrast, *An. farauti* had a biting peak in the early evening at 18.00–19.00 h with mostly outdoor biting, alongside a second biting peak at 01.00–02.00 h (Fig. 6d)^42,46^. The capture of human-seeking *Anopheles* at 18.00 h with immediate collections of specimens by volunteers points to the possibility of earlier biting and transmission. This evidence points to the need to characterize *Anopheles* biting earlier than the 18.00 h collection starting point^47^. ITN and IRS-based protections against these endophagic-exophilic adult *Anopheles spp.* will not be enough to eliminate malaria in this region^48^, with spatial and temporal gaps in protection allowing continued transmission.

Indoor resting data demonstrate that only a small proportion of vectors rest on walls pointing to the limited efficacy of IRS. This study demonstrates that evaluating and understanding the indoor resting rate of local vectors may be a vital component of the decision to implement this intervention an area. Areas with high indoor resting rates relative to the overall population (indoor resting rates versus overall biting rates) will have a higher IRS impact if the mosquitoes are susceptible to the insecticide. Provision of ITNs and IRS should be carefully assessed to optimize utility where appropriate^49^. Other tools, such as spatial and topical repellents, should be considered based on where and when exposure occurs. SBC may provide routine health awareness education to the community to mitigate the risk of malaria infection and minimize human-vector contact. Employing full-time assistant entomologists in each Primary Health Centre (PHC) to guide community-based vector control activities is essential for evidence-based decision-making.

One of the study outputs is the development of a map of the potential risk of malaria transmission in each study site. An example is shown in Fig. 7, the map from Skofro village, Keerom Regency, with the highest EIR value during this study. Skofro village is a small village (79.96 km^2^) with a population of only 149 persons, located close to the Papua New Guinea border^50^—a transit point with high cross-border mobility. Here, residential housing is mostly located parallel to the village's main road, as shown on the map. Examples of the *Anopheles* larval habitats are shown in Fig. 7. If we assume that adult *Anopheles* mosquitoes can fly about 500 m from their larval habitat^51^, we can draw the coverage of the potential risk of malaria transmission in the area, shown in the light red-circled area. Here, the residential area covered with red demonstrates that all households in the Skofro are accessible to adult *Anopheles* mosquitoes, where the darker red represents a higher potential for malaria transmission.

Larva source management, which includes habitat modification and manipulation, biological control, and larviciding, may be implemented to reduce mosquito density^52,53^. Here, the elimination of mosquito habitats, especially *Anopheles*, can be carried out together in the community, with local ownership, so that the effect is more pronounced. Vector-based malaria control may be performed by community members (community-driven) and guided by trained health personnel to reduce mosquito larval production within and surrounding dwellings and villages (Fig. 7).

This study has limitations because it was conducted cross-sectionally on eight sentinel sites. The result of breeding site observation, larval and pupal collection, HLCs, and adult resting behaviour may not represent the entire regency and geographic locations as reported previously^15,54^.

## Conclusions

This study confirmed that *Anopheles spp.* from the Punctulatus Group was abundant in all eight regencies in Papua. Three major species, namely *An. koliensis*, *An. punctulatus* and *An. farauti* were found in various ecosystems in Papua, Indonesia. These species play significant roles in malaria transmission indoors and outdoors. Therefore, this evidence explains why current malaria control efforts focusing on indoor protection fail to bring down malaria incidence in the region. Optimization of ITN usage, as well as instalment of mosquito screens, may further reduce indoor transmission. For prevention of outdoor transmission, the use of community-centric approaches to reduce or eliminate larval sources within and surrounding the village through the guidance of locally stationed entomologists, along with Social and Behavior Change (SBC) mediated health education towards the local adoption of mosquito protection tools during outdoor activities, may reduce malaria transmission.

### Supplementary Information

Supplementary Information 1.

Supplementary Information 2.

## Data Availability

All datasets generated and analyzed during this study are included in the manuscript.
